# Decreased IGF1R attenuates senescence and improves function in pancreatic β-cells

**DOI:** 10.3389/fendo.2023.1203534

**Published:** 2023-06-27

**Authors:** Kanako Iwasaki, Benjamin Lalani, Jiho Kahng, Priscila Carapeto, Stephanie Sanjines, Francesko Hela, Cristian Abarca, Tadataka Tsuji, Justin Darcy, Andrzej Bartke, Yu-Hua Tseng, Rohit N. Kulkarni, Cristina Aguayo-Mazzucato

**Affiliations:** ^1^Section on Islet Cell Biology and Regenerative Medicine, Joslin Diabetes Center and Harvard Medical School, Boston, MA, United States; ^2^Medical Research Institute, Kitano Hospital, Osaka, Japan; ^3^Section on Integrative Physiology and Metabolism, Joslin Diabetes Center and Harvard Medical School, Boston, MA, United States; ^4^Department of Internal Medicine, Division of Geriatrics Research, Department of Medicine, Southern Illinois University School of Medicine, Springfield, IL, United States

**Keywords:** senescence, SASP, IGF-1, insulin, Dwarf, diabetes mellitus, β-cell

## Abstract

**Introduction:**

The enhanced β-cell senescence that accompanies insulin resistance and aging contributes to cellular dysfunction and loss of transcriptional identity leading to type 2 diabetes (T2D). While senescence is among the 12 recognized hallmarks of aging, its relation to other hallmarks including altered nutrient sensing (insulin/IGF1 pathway) in β-cells is not fully understood. We previously reported that an increased expression of IGF1R in mouse and human β-cells is a marker of older β-cells; however, its contribution to age-related dysfunction and cellular senescence remains to be determined.

**Methods:**

In this study, we explored the direct role of IGF1R in β-cell function and senescence using two independent mouse models with decreased IGF1/IGF1R signaling: a) Ames Dwarf mice (Dwarf ^+/+^), which lack growth hormone and therefore have reduced circulating levels of IGF1, and b) inducible β-cell-specific IGF1R knockdown (βIgf1rKD) mice.

**Results:**

Compared to Dwarf^+/-^ mice, Dwarf^+/+^ mice had lower body and pancreas weight, lower circulating IGF1 and insulin levels, and lower IGF1R and p21Cip1 protein expression in β-cells, suggesting the suppression of senescence. Adult βIgf1rKD mice showed improved glucose clearance and glucose-induced insulin secretion, accompanied by decreased p21Cip1 protein expression in β-cells. RNA-Seq of islets isolated from these βIgf1rKD mice revealed the restoration of three signaling pathways known to be downregulated by aging: sulfide oxidation, autophagy, and mTOR signaling. Additionally, deletion of IGF1R in mouse β-cells increased transcription of genes important for maintaining β-cell identity and function, such as *Mafa*, *Nkx6.1*, and *Kcnj11*, while decreasing senescence-related genes, such as *Cdkn2a*, *Il1b*, and *Serpine 1*. Decreased senescence and improved insulin-secretory function of β-cells were also evident when the βIgf1rKD mice were fed a high-fat diet (HFD; 60% kcal from fat, for 5 weeks).

**Discussion:**

These results suggest that IGF1R signaling plays a causal role in aging-induced β-cell dysfunction. Our data also demonstrate a relationship between decreased IGF1R signaling and suppressed cellular senescence in pancreatic β-cells. Future studies can further our understanding of the interaction between senescence and aging, developing interventions that restore β-cell function and identity, therefore preventing the progression to T2D.

## 1 Introduction

Pancreatic β-cells play a critical role in glucose homeostasis by secreting insulin in response to a rise in glycemia that stimulates glucose uptake by peripheral tissues. The insulin secretion capacity of individual β-cells increases as a form of compensation in response to insulin resistance in target metabolic tissues. Type 2 diabetes (T2D) is an age-related disease characterized by a decrease in β-cell mass and function with a failure to compensate for the high insulin demand during insulin resistance (1). Aging is marked by the loss of homeostasis and functional decline in tissues, which correlates with age-related diseases such as T2D. At a cellular level, aging is driven by 12 hallmarks that fulfill three criteria: 1) their manifestation associates with age, 2) they are accentuated by experimental models of aging acceleration, and 3) it is possible to decelerate, stop, or reverse them through therapeutic interventions (2). Two of these hallmarks are altered nutrient sensing acting *via* the insulin/insulin-like growth factor-1 (IGF1) pathway and cellular senescence. Although it is recognized that the distinction among these hallmarks is diffused because they interact and are not independent of each other, much remains to be done in terms of how they relate to specific cell types and disease processes.

We previously identified increased IGF1R as a marker of older β-cells in human donors older than 40 years and mice older than 1 year (3). Even in donors younger than 40 years with T2D, increased IGF1R positivity (IGF1R+) in β-cells is associated with higher expression levels of *p16Ink4a* mRNA levels and increased senescence-associated β-galactosidase (SA-βGal) activity, consistent with them being senescent. Additionally, high-intensity IGF1R+ β-cells were dysfunctional, characterized by a lack of glucose responsiveness.

In aged, insulin-resistant, and T2D mice, insulin-secreting pancreatic β-cells became senescent, characterized by upregulation of senescence markers: p21^Cip1^, p16^Ink4a^ (4), increased activity of SA-βGal, and increased transcription and secretion of the senescence-associated secretory phenotype (SASP) like IL1α and IL6 (4). Cellular senescence contributed to cellular dysfunction and loss of transcription identity leading to T2D. Interventions that decreased the load of senescent cells (senolytics) improved β-cell function, their transcriptional profile, and blood glucose (5). These results suggest that increased levels of IGF1R are related to dysfunction and senescence in β-cells. However, the direct correlation between increased expression of IGF1R and senescence in β-cells has never been directly tested. In addition, it remains unknown whether high IGF1R is only a marker of older β-cells or an effector of age-related changes in function and identity.

In this study, we used two mouse models with decreased IGF1/IGF1R signaling: a) Ames Dwarf (*Prop1^df^/Prop1^df^*) mice (Dwarf^+/+^), which have reduced circulation levels of IGF1 due to growth hormone deficiency (6, 7), and b) inducible β-cell-specific IGF1R knockdown (βIgf1rKD) mice. In both models, attenuation of the IGF1R signaling pathway suppressed cellular senescence and improved glucose tolerance. These results underscore the critical role of the IGF1R signaling pathway in aging-induced β-cell senescence and dysfunction.

## 2 Materials and methods

### 2.1 Animals

Ames Dwarf (*Prop1^df^/Prop1^df^*) mice derived from breeders were obtained from Southern Illinois University School of Medicine and described previously (7) and obtained from Dr. Michal Masternak at the University of Central Florida. All experiments were conducted at Joslin Diabetes Center with the approval of its Animal Care and Use Committee; mice were kept on a 12-h light/12-h dark cycle and had free access to water and food (LabDiet #5020; fat 21.6%, protein 23.2%, and carbohydrates 55.2%). The body weight, pancreas weight, blood glucose (BG), insulin, and IGF-1 were measured after 4 h of fasting at the age of 9–34 months in 18 Dwarf^+/+^ and 11–14 months in nine Dwarf^+/−^ mice.

We used a mouse model in which *Igf1r* is specifically decreased in β-cells by crossing mice floxed for exon 3 of the *Igf1r* gene with mice expressing tamoxifen-inducible Cre recombinase under a rat insulin promoter (8, 9). Tamoxifen (Sigma-Aldrich, St. Louis, MO, USA; #10540-29-1) was dissolved by corn oil (Sigma; C8267) and administered *via* intraperitoneal injection (0.075 mg/g body weight) once every 24 h for five consecutive days to generate βIgf1rKD (Ins1-CreERT2^+/−^ Igf1r^flox/flox^) mice. For comparison, βIgf1r (Ins1-CreERT2^−/−^ Igf1r^flox/flox^) mice were treated with corn oil. Seven days after the final injection, islets were isolated and analyzed. To create an insulin resistance model, βIgf1rKD mice and littermate controls were fed a high-fat diet (Rodent Diet #D12492; fat 60 kcal%, protein 20%, and carbohydrate 20%) (HFD-βIgf1rKD) for a period of 5 weeks, which has been shown to induce insulin resistance in mice (10) and accelerate the appearance of senescent β-cells (5). Tamoxifen was administered during the first 5 days of the high-fat diet (HFD). Also, to test the effects of tamoxifen treatment in non-transgenic mice, 8–12-month-old male C57Bl/6 mice were used with and without treatment. All experiments included males and females, except where stated.

### 2.2 Quantitative reverse-transcription PCR

Quantitative reverse-transcription PCR (qRT-PCR) was carried out as previously described (5). Briefly, total RNA isolated with RNEasy Plus Mini Kit (QIAGEN, Valencia, CA, USA) was reverse transcribed (SuperScript reverse transcriptase; Invitrogen, Carlsbad, CA, USA). SYBR green (Thermo Scientific, Waltham, MA, USA; #A25741) and specific primers were used to detect the expression of specific genes. Expression levels were normalized using *Actb* as an internal control, and the comparative delta-delta threshold cycle (ΔΔCt) method was used to calculate relative gene expression levels. The sequences of each primer are listed in **Supplementary Table 1**.

### 2.3 Immunohistochemistry

Immunostaining of the pancreas in the Dwarf^+/+^, Dwarf^+/−^, βIgf1rKD, and βIgf1r mice was performed as described (5). Briefly, paraffin sections were deparaffinized with ethanol gradients, followed by washing with phosphate-buffered saline (PBS) and antigen retrieval with citric acid and permeabilization with a Triton X 0.3% solution. After washing with PBS and blocking with 1% normal donkey serum (NDS), the slides were incubated overnight with primary antibody IGF1R (1:100; sc-713 Rbt anti-Igf1rβ), INSULIN (1:400; Bio-Rad; 5330-0104G), and P21CIP1 (1:100; Cell Signaling, Danvers, MA, USA; 2947). Biotin-streptavidin amplification was used for IGF1R detection. Following subsequent washes, the slides were incubated overnight with a primary anti-insulin antibody. Incubations for 1 h with a secondary antibody for insulin were coupled with DAPI for nuclear staining. For each antibody, sections were stained and imaged in parallel such that the staining intensity reflected protein expression. For quantification, images were captured systematically covering the whole section in confocal mode on a Zeiss LSM 710 microscope.

### 2.4 Quantification of protein expression in Dwarf^+/+^ and Dwarf^+/−^ mice

Paraffin sections from 7-month-old male Dwarf^+/+^ and Dwarf^+/−^ mice were stained in parallel and analyzed using confocal microscopy. Between 899 and 2,739 cells from 10 islets were counted from five animals per condition stained for IGF1R, INSULIN, and P21CIP1. The imaging settings were kept consistent between samples to allow for comparison of protein concentration based on intensity.

### 2.5 Intraperitoneal glucose tolerance tests

Glucose tolerance tests were conducted after 6 h of fasting. Blood samples were collected from the tails at 0, 15, 30, 60, 90, and 120 min after an intraperitoneal injection of glucose (2 g/kg body weight). For *in vivo* glucose-stimulated insulin secretion (GSIS), the insulin levels were measured from serum collected at the 0- and 15-min time points of the intraperitoneal glucose tolerance test (IPGTT). Four mice in each group were used for IPGTT in βIgf1rKD and βIgf1r. In the HFD study, seven mice were tested in the following groups: HFD-βIgf1rKD, HFD-βIgf1r, and Chow-βIgf1r. The serum levels of mouse insulin (Mercodia, Winston Salem, NC, USA; 10-1247-01) and Igf-1 (Invitrogen; EMIGF1) were measured using ELISA kits.

### 2.6 Islet isolation

Islets were isolated by injecting collagenase Vitacyte (Indianapolis, IN, USA; CIzyme RI; 005-1030) into the pancreatic duct, followed by digestion in a 37°C water bath. After several washes with Medium199 (Thermo Scientific; 21180021) with 10% newborn calf serum (Sigma-Aldrich; N4762), islets were filtered through a 425-µm-diameter wire mesh (Cole-Parmer, Vernon Hills, IL, USA; SI59987-16) and collected by gradient separation with Lymphocyte Separation Medium (Corning, Glendale, AZ, USA; 25-072-CV). Islets from individual animals were kept separate and analyzed by qPCR or RNA-seq.

### 2.7 Chemicals and reagents

All Western blotting buffers and reagents were purchased from Bio-Rad (CA, USA). Anti-BETA ACTIN (Cat no. ab8226) antibody was purchased from Abcam (Cambridge, UK). Anti-IGF-I Receptor β (Cat no. 9750), anti-P21 (Cat no. 2947), and anti-PDX1 (Cat no. 5679) antibodies were obtained from Cell Signaling Technologies (MA, USA). The dilutions of all primary antibodies were 1:1,000.

### 2.8 Western blotting

Proteins were extracted from mouse islets using a 10X radioimmunoprecipitation assay buffer (Cell Signaling Technologies, MA, USA). All the mice used in Western blotting were male and female aged 4–9 months, with five mice per group. The concentration of the samples was quantified using bicinchoninic acid (BCA) assay. Equal amounts of protein for every sample (20 µg) were run on sodium dodecyl sulfate–polyacrylamide gel electrophoresis (SDS-PAGE) and transferred onto an Immuno-Blot polyvinylidene difluoride (PVDF) membrane using Bio-Rad wet transfer system. The blocking step was performed for 1 h at room temperature using 5% non-fat dry powder milk in TBST (Bio-Rad, CA, USA). After the blocking step, the membranes were incubated with the respective diluted primary antibodies overnight at 4°C. Following three washing steps, the blots were incubated with horseradish peroxidase (HRP)-conjugated secondary antibodies for 1.5 h at room temperature. After three final washing steps, membranes were incubated with enhanced chemiluminescence (ECL) for 2 min, and the signals on the blots were visualized using ChemiDoc MP Imaging System (Bio-Rad, CA, USA).

### 2.9 Bulk RNA-seq

Islets were isolated from four βIgf1rKD and four βIgf1r mice aged 17 months, including both genders in each group. The islets were resuspended in an RNA extraction buffer, and the RNeasy Micro Kit (No. 74004) was used to purify RNA and submitted for sequencing to DNA Link (Los Angeles, CA, USA). Data analysis was performed by the Bioinformatics and Biostatistics Core at Joslin Diabetes Center. The sequencing data have been deposited into Gene Expression Omnibus (GEO) from National Center for Biotechnology Information (NCBI) (accession GSE229709).

### 2.10 Statistical analysis

The statistical analysis was performed by using Graph Pad Prism. All the data were determined to be consistent with a normal distribution, and measurements were compared by t-test or one-way ANOVA followed by post-hoc Tukey’s test. The differences of p < 0.05 were considered to be statistically significant. In all the data, *p < 0.05, **p < 0.01, ***p < 0.001, and ****p < 0.0001.

## 3 Results

### 3.1 Phenotype of Ames Dwarf (*Prop1^df^*/*Prop1^df^*) mice

**Figure 1:**
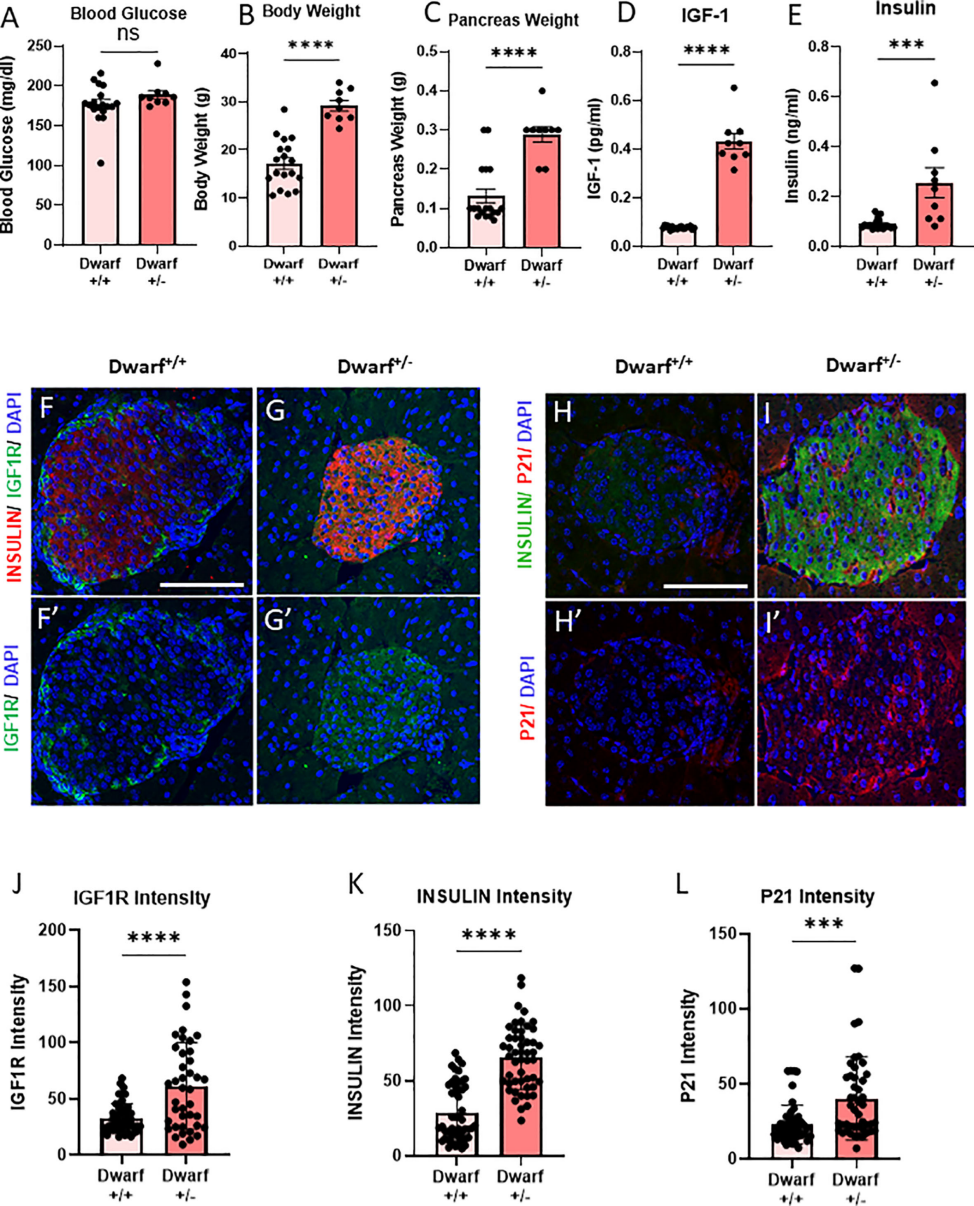
Decreased INS/IGF1R in β-cells from Dwarf^+/+^ was accompanied by decreased senescence marker P21^CIP1^. **(A–C)** Differences in blood glucose, body weight, and pancreas weight between Dwarf^+/+^ (10 male/8 female, 11–14/9–34 months) and Dwarf^+/−^ (5 male/4 female, 11–14/12–14 months) mice. **(D, E)** Circulating IGF1 and insulin levels between Dwarf^+/+^ and Dwarf^+/−^ mice. **(F–I′)** Immunostaining of islets from Dwarf^+/+^ (5 males at 11–13 weeks) and Dwarf^+/−^ (5 males at 11–14 weeks) mice showing insulin (red/green) in islets, DNA stained with DAPI (blue), IGF1R **(F–G′)** in green, and P21 **(H–I′)** in red. The white scale bars represent 100 μm. Image analysis to quantify the intensity differences of IGF1R **(J)**, INSULIN **(K)**, and P21CIP1 **(L)** between islets from Dwarf^+/+^ and Dwarf^+/−^ mice, using t-test, with ***p < 0.001 and ****p < 0.0001, respectively. ns, not significant.

To evaluate the differences between Ames Dwarf (*Prop1^df^*/*Prop1^df^*) (Dwarf^+/+^) mice and their heterozygous littermates (Dwarf^+/−^), we measured body and pancreas weight, and blood glucose levels after 4 h of fasting. There was no significant difference in fed blood glucose levels between Dwarf^+/+^ and Dwarf^+/−^ mice (**Figure 1A**). In Dwarf^+/+^, body weight was decreased by 40%, while pancreatic weight was decreased by 50% (**Figures 1B, C**). Consistent with decreased pituitary growth hormone axis, Dwarf^+/+^ mice had significantly lower circulating IGF-1 and insulin levels than Dwarf^+/−^ mice (**Figures 1D, E**). There were no gender differences in these parameters (**Supplementary Figures 1A–H**); therefore, the results are shown for both sexes combined.

### 3.2 Immunochemistry showed a decreased number of P21-positive β-cells in Dwarf^+/+^ mice

To understand the effects of the Dwarf^+/+^ genotype on β-cell IGF1R protein levels, pancreatic slides were stained and quantified for INSULIN and IGF1R. There was a 40% decrease in IGF1R protein intensity in the Dwarf^+/+^ islets (**Figures 1F–G′, J**). Interestingly, INSULIN protein intensity levels in islet β-cells were also decreased by 40% (**Figure 1K**). These results are consistent with the downregulation of the IGF1R expression in β-cells. To examine the effects of this downregulation on senescence, the same sections were stained for P21CIP1, a marker and effector of senescence, which showed a significant decrease of 50% in β-cells (**Figures 1H–I′, L**). These results are consistent with decreased β-cell senescence in a genetic mouse model of decreased IGF1R in the same cell type. Dwarf**^+/+^** mice are a well-accepted and recognized model of longevity and delayed aging where the participation of the IGF1 pathway is well established (11, 12). However, these mice are global knockout that also lack other pituitary hormones (prolactin and thyroid stimulatory hormone). Therefore, to dissect the specific mechanistic role of IGF1R in β-cell senescence, we developed a tissue-specific conditional deletion model.

### 3.3 Improvement of glucose clearance and glucose responsiveness in β-cell-specific Igf1r knockdown (βIgf1rKD) mice

**Figure 2:**
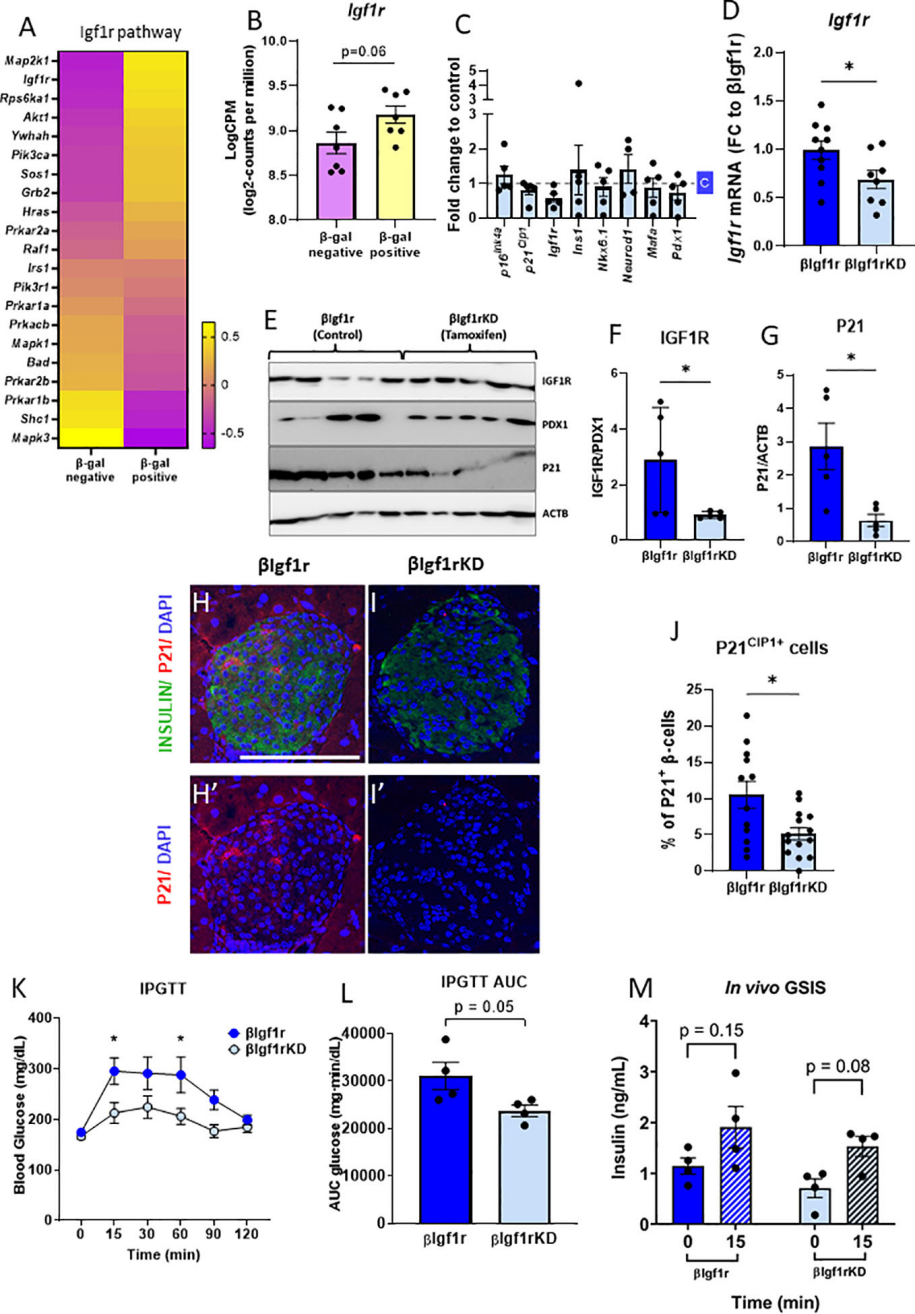
βIgf1rKD mice showed improved glucose clearance and glucose-induced insulin secretion, accompanied by decreased p21Cip1 gene and protein expression in β-cells. RNA-seq from senescent (βGal-positive) and non-senescent (βGal-negative) islet cells revealed an upregulation of the IGF1R pathway **(A)** and increased Igf1r expression **(B)** in senescent β-cells of C57Bl/6 mice. **(B)** Read counts were converted to log2-counts per million (LogCPM). In the β-cell-specific model of downregulation of Igf1r, all the bars with dark blue represent βIgf1r and with light blue βIgf1rKD. **(C)** mRNA expression by qPCR comparing gene expression in islets from C57Bl/6 tamoxifen-treated mouse islets, normalized to a vehicle **(c)**-treated mice. **(D)** The mRNA level of *Igf1r* in islets of βIgf1r and βIgf1rKD mice; male and female at the age of 11–19 months, with eight to 10 mice per group. **(E–G)** IGF1R and P21 levels in βIgf1rKD and βIgf1r mouse islets from the Western blotting were normalized by PDX1, which is only expressed in β-cells in mature islets, and ACTB, respectively. Male and female at the age of 4–9 months, with five mice per group. Representative pictures of stained islets from one male mouse each in βIgf1r **(H, H′)** and βIgf1rKD group **(I, I′)**, at the age of 16.5 months. INSULIN in green, P21 in red, and DAPI in blue. The scale bars represent 100 μm. **(J)** The percentage of P21CIP1 positive cells in β-cells in βIgf1r and βIgf1rKD mice. **(K–M)** Intraperitoneal glucose tolerance test (IPGTT) to βIgf1r and βIgf1rKD mice, four animals per condition at the age of 17 months, including both male and female. Blood glucose **(K)**, the area under the curve (AUC) of their glucose levels **(L)**, and insulin levels **(M)**. The insulin concentration was measured from serum collected at the 0- and 15-min time points after the glucose injection. Using t-test, *p < 0.05.

To understand whether senescent β-cells had increased levels of Igf1r, our previously published RNA-seq data (5) from senescent (βGal-positive) and non-senescent (βGal-negative) cell populations of mouse β-cells, were queried for *Igf1r* levels and pathway analysis. Pathway analysis revealed a significant decrease in the IGF1 pathway in the βGal-negative group compared to the βGal-positive group (**Figure 2A**). The levels of *Igf1r* were lower in the βGal-negative group (**Figure 2B**). These results support higher levels of IGF1R in senescent β-cells.

To specifically test the role of IGF1R in β-cell function, identity, and senescence, a conditional mouse model to knockdown IGF1R in β-cells was developed and induced by tamoxifen administration. To control for the acute and direct effects of tamoxifen treatment in islets, male C57Bl/6 mice aged 8–12 months were treated with the drug and with the vehicle. There were no significant differences in gene expression of key β-cell genes (**Figure 2C**), consistent with previous reports of a lack of effects of tamoxifen administration on glucose homeostasis (13, 14).

When βIgf1rKD (Ins1-CreERT2^+/−^ Igf1r^flox/flox^) mice were treated with tamoxifen, there was a 30% reduction of *Igf1r* mRNA levels when compared to βIgf1r (Ins1-CreERT2^−/−^ Igf1r^flox/flox^) (p < 0.05) (**Figure 2D**). IGF1R protein expression levels were decreased by 69% in islets of βIgf1rKD compared to βIgf1r (**Figures 2E, F**). These results were normalized to PDX1, which is only expressed in β-cells and therefore excludes potentially confounding results coming from IGF1R expression from other islet cell types, such as α, δ, or PP cells. In addition, P21CIP1 expression was significantly reduced in the islets of βIgf1rKD compared to that of the βIgf1r mice (**Figures 2E, G**). We also performed immunostaining of pancreas sections for P21CIP1 and insulin, which showed a significant decrease in their expression levels in βIgf1rKD compared to βIgf1r mice (**Figures 2H–J**). Consistent with decreased senescence when IGF1R is downregulated, P21CIP1 levels were decreased by 50% in β-cells in the islets from βIgf1rKD mice (**Figure 2J**).

To understand the effects of decreased IGF1R levels on β-cell function and glucose homeostasis, we performed IPGTT in βIgf1rKD and βIgf1r mice. βIgf1rKD showed significantly lower glucose levels at 15 and 60 min after glucose injection, also seen in the area under the curve (AUC), consistent with improved glucose clearance (**Figures 2K, L**). Additionally, *in vivo* evaluation of GSIS revealed a loss of glucose responsiveness in βIgf1r mice, which tended to be restored after decreasing levels of Igf1r in β-cells (**Figure 2M**). These results suggest that the downregulation of Igf1r in β-cells decreases senescence, potentially improving glucose homeostasis.

### 3.4 Decreased expression of senescence and SASP genes with improved β-cell identity genes in islets from βIgf1rKD mice

**Figure 3:**
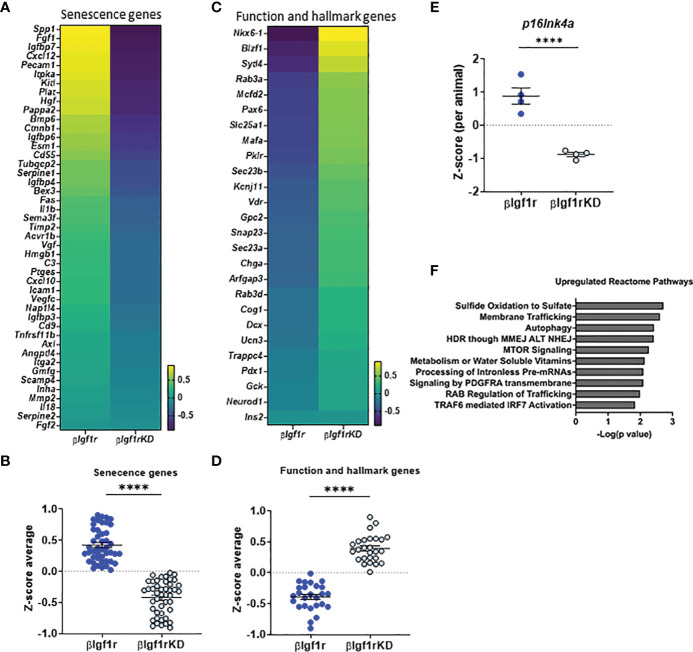
RNA-seq from islets with downregulation of Igf1r revealed decreased markers of senescence and increased expression of β-cell identity genes. Heatmaps and Z-score average expression data from RNA-seq of islets of βIgf1rKD and βIgf1r mice showing changes in senescence signature genes **(A, B)** and key β-cell function and hallmark genes **(C, D)**. Differential gene expression of *p16^Ink4a^* **(E)** per animal. **(F)** Reactome pathway analysis of genes differentially expressed in βIgf1rKD and βIgf1r. Male and female at the age of 17 months, with four mice per group, ****p < 0.0001.

To understand the overall transcriptional changes in islets from βIgf1rKD, bulk RNA-seq analysis was performed and showed that 945 genes out of 14,806 were differentially expressed between the βIgf1rKD and βIgf1r groups. Since there are no specific universal senescent markers that are valid across all cell types and tissues, it is recommended that a combination of markers be used to identify senescent cells. Analysis of senescence and SASP factor gene expression included a subset of the recently published SenMayo panel (15) (**Figures 3A, B**), which showed overall downregulation in islets from βIgf1rKD mice. The expression level of the *p16^Ink4a^*, a known senescence marker and effector, was significantly decreased in βIgf1rKD mice compared to that of the control (**Figure 3E**). βIgf1rKD mice showed increased expression of β-cell function and hallmark genes when compared to βIgf1r mice (**Figures 3C, D**), consistent with improved glucose clearance and *in vivo* GSIS. Additionally, pathway analysis revealed increased expression of three pathways that are known to go down with aging: sulfide oxidation to sulfate, autophagy, and mTOR signaling (**Figure 3F**). Specific gene analysis of the autophagy pathway (**Supplementary Figures 2A, B**) revealed the upregulation of many of these genes. Given that autophagy is among the most important protective catabolic processes in the cells activated when the cells experience different stressors that disturb homeostasis, it is suggested that IGF1R in β-cells might be interacting with other hallmarks of aging (autophagy) in addition to senescence. Further, Reactome pathway analysis for the RNA-seq of the two groups, βIgf1r and βIgf1rKD, revealed two additional and relevant upregulated pathways when Igf1r is knocked down: G2-M DNA damage checkpoint and DNA double-strand break response (**Supplementary Figures 2C–F**), both of which are directly connected to senescence. Collectively, these data suggest that conditional downregulation of Igf1r in β-cells in adulthood ameliorated cellular senescence through the enhancement of DNA damage mechanisms. Additionally, it decreased markers of senescence and increased functional and hallmark genes in the cells that are potentially primed to undergo senescence, as shown by a recovery of their transcriptional identity and decreased expression of senescence genes.

### 3.5 Improved β-cell transcriptional identity in βIgf1rKD mice on HFD

**Figure 4:**
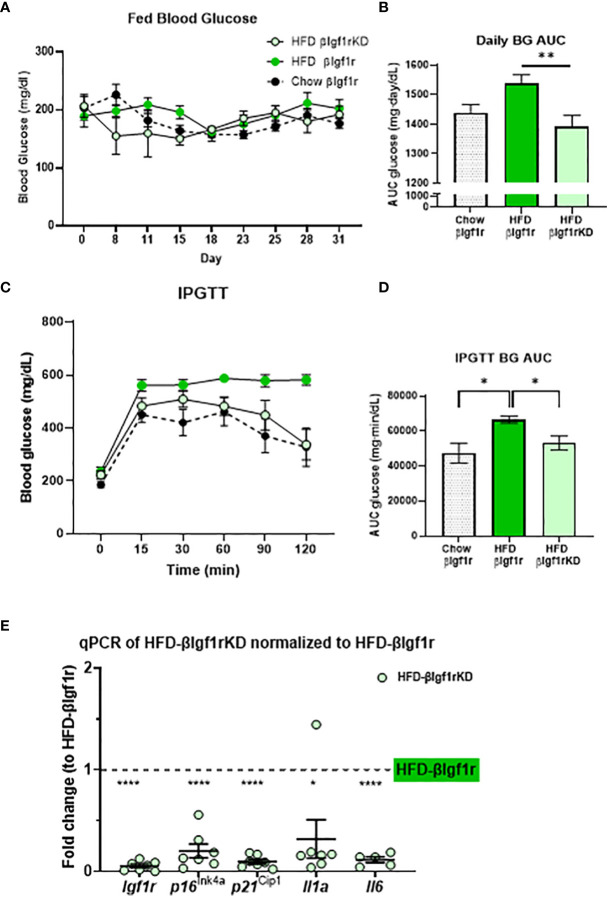
In a model of a high-fat diet, downregulation of Igf1r in β-cells improved glucose levels and decreased expression of senescence and senescence-associated secretory phenotype (SASP) genes. Fed blood glucose levels and area under the curve (AUC) after 5 weeks of a high-fat diet in βIgf1rKD (HFD-βIgf1rKD) in light green, βIgf1r (HFD-βIgf1r) in green, and normal chow in βIgf1r mice in dots **(A, B)**. Intraperitoneal glucose tolerance test (IPGTT) results of blood glucose levels **(C)** and its AUC **(D)**. The mRNA expression of *Igf1r*, *p16^ln4a^*, *p21^Cip1^*, *Il1a*, and *Il6* in HFD-βIgf1rKD compared with βIgf1r under the same HFD condition **(E)**. HFD-βIgf1rKD (5 male/2 female, 3–12 months), HFD-βIgf1r (5 male/2 female, 3–8 months), and normal chow in βIgf1r (6 male/1 female, 3–8 months), using t-test or one-way ANOVA, with *p < 0.05, **p < 0.01, and ****p < 0.0001, respectively.

The induction of senescence in β-cells and adipose tissue by HFD has been previously shown by us and others (5, 16–18). Herein, mice were fed HFD for 5 weeks to evaluate the effect of βIgf1r knockdown in an adult model of insulin resistance (which is known to increase β-gal activity) and transcription of senescence and SASP genes. Tamoxifen or vehicle was administered during the first 5 days of HFD to induce knockdown of the Igf1r in β-cells. Since the effects of βIgf1r knockdown in the chow diet are shown in **Figures 2, 3**, this section only compared the knockdown group during HFD. During the 5 weeks, the AUC of fed glucose level in HFD fed βIgf1rKD (HFD-βIgf1rKD) was significantly lower than that in HFD fed βIgf1r (HFD-βIgf1r) (**Figures 4A, B**). HFD-βIgf1rKD mice had improved glucose clearance during IPGTT (**Figures 4C, D**). During the first 10 days, body weight was decreased in the HFD-βIgf1rKD when compared to HFD-βIgf1r (**Supplementary Figures 3A, B**); however, there were no differences in the food consumption measured between days 11 and 15 (**Supplementary Figures 3C, D**). This initial weight loss might have been caused by nausea, which is known to be induced by tamoxifen treatment, which preceded the switch in diet (19).

In the HFD group, βIgf1rKD led to a significant downregulation in the expression of senescence and SASP genes in pancreatic islets: *p16lnk4a*, *p21Cip1*, *Il1a*, and *Il-6* (**Figure 4E**). The results of the βIgf1rKD experiment under a normal chow are shown in **Figure 2**.

**Figure 5:**
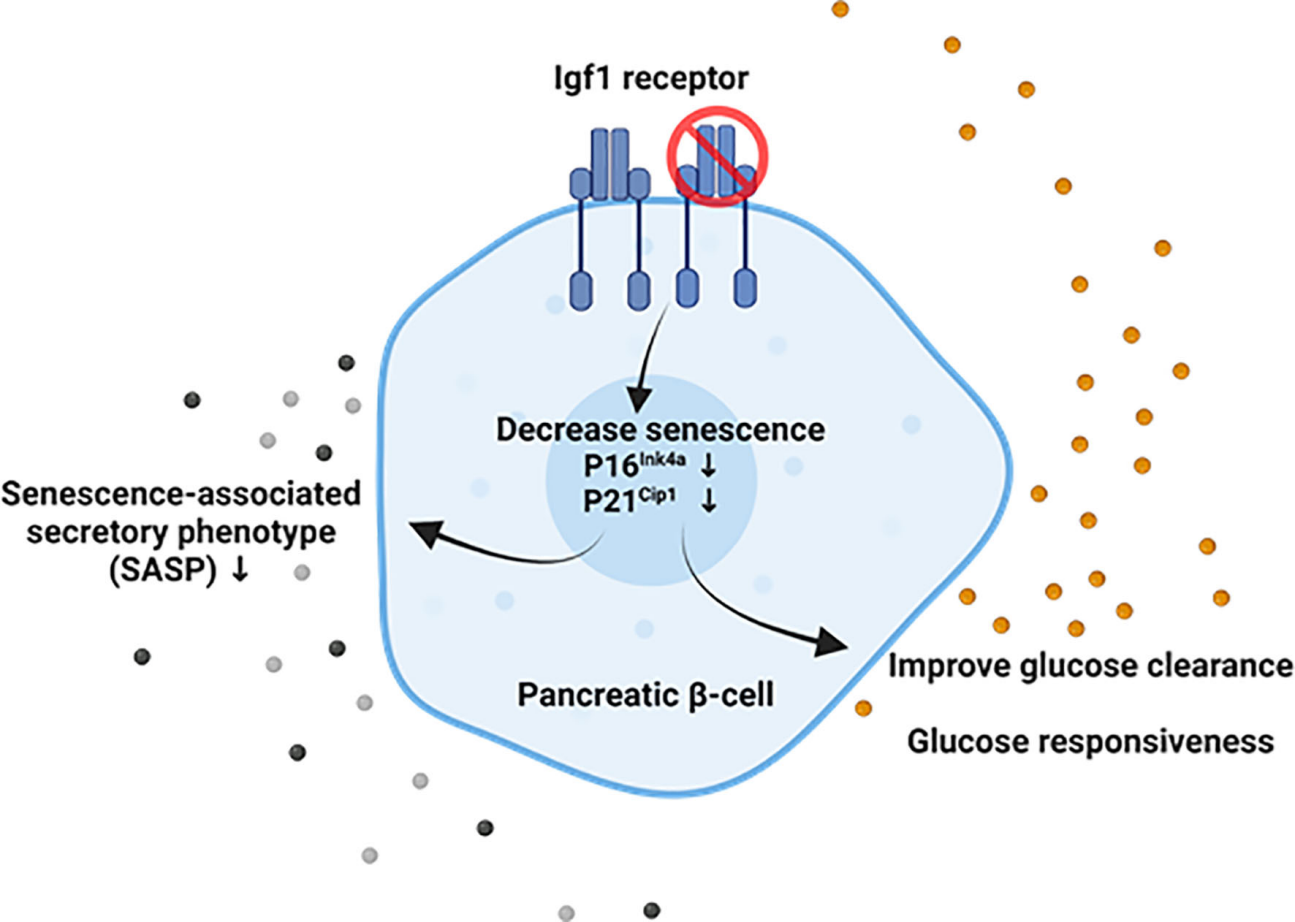
Suggested model of the interaction between Igf1r and senescence in adult mouse β-cells. Our results suggest that downregulation of Igf1r in senescent β-cell leads to decreased p16 gene expression and p21 protein levels, both markers and effectors of senescence, decreased gene expression of senescence-associated secretory phenotype (SASP) factors in a model of a high-fat diet, and improved glucose clearance and restored loss of glucose responsiveness compared to βIgf1r (control) in intraperitoneal glucose tolerance test (IPGTT). Created with BioRender.com.

Overall, these results show that the downregulation of Igf1r in both global and β-cell-specific mouse models is associated with decreased β-cell senescence, improved function, and cellular identity (**Figure 5**). This establishes a link between two hallmarks of aging, cellular senescence and deregulated nutrient-sensing, which might be relevant for the loss of cell identity and increased senescence during diabetes progression.

## 4 Discussion

Igf1r expression is increased in β-cells from older mice and humans (3), which are also known to accumulate senescence characteristics (5). However, it is unknown whether manipulation of the Igf1/Igf1r pathway can alter senescence in β-cells. In this study, we demonstrated that attenuation of the Igf1/Igf1r pathway in adult mice led to decreased expression of senescence markers and improved function of β-cells. These changes were accompanied by improved glucose tolerance *in vivo*, supporting the importance of this pathway as a therapeutic target in T2D.

We employed two murine models of the Igf1/Igf1r pathway attenuation: Ames Dwarf mice and β-cell-specific *Igf1r* knockdown mice (βIgf1rKD). Ames Dwarf mice have no growth hormone (GH) signaling with suppressed circulating IGF-1. Dwarf mice are homozygous for the Prop1^df^ mutation and live an average of 49% to 64% longer than control littermates (6, 7). Published studies have reported that Dwarf mice have decreased levels of Igf1r in the brain (20). Accordingly, we detected lower IGF1R levels in β-cells, which correlated with decreased expression of senescence markers. These results suggest that suppression of the Igf1/Igf1r pathway protects mice from β-cell senescence. Ames Dwarf mice are viable and long-lived, which is reminiscent of human patients with decreased activity of the GH–IGF-I axis. In humans, Laron syndrome, caused by the mutation of the growth hormone receptor (GH-R) gene, is characterized by a remarkable degree of protection from age-related diseases such as cancer and diabetes (21, 22). Oral glucose tolerance tests (OGTTs) in Laron syndrome showed slightly higher insulin secretion from the fasting level than the control (normal) group, but defective insulin secretion after glucose load leads to hyperglycemia when compared to controls (23). Notwithstanding their obesity, only nine (eight men) out of 75 patients developed T2D (24). However, patients with acromegaly, known to have increased GH secretion, have a high incidence of diabetes, cancer, and heart disease and reduced life expectancy (25). Interestingly, patients surgically treated for acromegaly who achieved a reduction in serum IGF-1 but not GH levels showed improved glucose tolerance in the OGTT during the early postoperative period (26). These results strongly support an inverse correlation between IGF1 activity and glucose homeostasis in adulthood.

The other model used herein of Igf1/Igf1r pathway attenuation was an inducible β-cell-specific knockdown of Igf1r (βIgf1rKD: Ins1-Cre^ERT2+/−^ Igf1r^flox/flox^). These mice showed decreased expression of β-cell senescence genes and improved glucose tolerance and GSIS. These results were surprising given previous publications showing that a constitutive β-cell-specific knockout of Igf1r in mice had defective GSIS and impaired glucose tolerance due to reduced expression of *Slc2a2* (also known as Glut2) and *Gck* (encoding glucokinase), which couple glucose stimulus to insulin secretion (8, 27). Potential reasons for these discordant results are that in our model, the signaling downregulation occurs in adulthood when it is known that this pathway can mediate some age-related cellular damage. Another possibility is that in our model, we only obtain a 30% reduction in Igf1r transcription instead of complete knockout in the previous studies. This supports a model in which Igf1r signaling is required during the growth and development of β-cells for proper function and maturation but that an age-related increase in its abundance carries a higher risk for the development of a senescent phenotype. In accordance with this concept, we previously showed (3) that cells with high levels of IGF1R increase three-fold in old (1.7–2-year-old) mice compared to young (3–7-month-old) mice. These high IGF1R+ cells had higher levels of *p16Ink4a*, a marker and effector of senescence, and higher levels of *Ldha*, indicating β-cell dedifferentiation.

β-cell senescence is not always deleterious. It has been reported that induction of p16Ink4a under the *Ins* or *Pdx1* promoters in β-cells of 3–4-week-old transgenic mice induced senescence markers and β-cell function (28). At this age, pancreatic β-cells are not fully matured from a functional point of view, and therefore accelerating cellular aging through senescence might also bring a functional phenotype characteristic of older mature cells.

Taken together, these findings support the beneficial role of downregulating the insulin/IGF-I signaling pathway in extending lifespan and health span in diverse species, including yeast, worms, fruit flies, and rodents (29). In addition, downregulation of the insulin/IGF1R pathways has been correlated with increased longevity, and a mutation in the human *IGF1R* gene was detected in a study of centenarians (30). Furthermore, this pathway is directly downregulated by caloric restriction and has well-described metabolic benefits. Short-term IGF-1 induces cell proliferation that is antagonized by p53; however, it has been shown that prolonged IGF-1 treatment leads to premature cellular senescence by activating the tumor suppressor protein p53 (31). Disruption of the IGF1 pathway reduces senescence after IGF-1 treatment.

Future studies on the interplay of aging features will shed light on the development of specific interventions that restore β-cell function and identity during the progression to type 2 diabetes.

## Data availability statement

The data presented in the study are deposited in the Gene Expression Omnibus (GEO) from the National Center for Biotechnology Information (NCBI). Accession number GSE229709.

## Ethics statement

All experiments were conducted at Joslin Diabetes Center with the approval of its Animal Care and Use Committee under protocol number 2022-01. No human samples or data were used in this manuscript.

## Author contributions

CA-M contributed to the conception and design of the study and organized the database. KI, BL, JK, PC, SS, FH, and CA-M performed the statistical analysis. KI and CA-M wrote the first draft of the manuscript. KI, BL, JK, FH, and CA-M wrote sections of the manuscript. RK provided the βIgf1r flox mouse model and advice on the experimental design. YH-T, AB, JD, and TT provided the Dwarf mice and expertise with experiments and sample handling. All authors contributed to the article and approved the submitted version.

## Supplementary material

Supplementary Figure 1 Related to **(A-F)** The blood glucose level at 4 hours fast, body and pancreas weight were compared between Dwarf^+/+^ and Dwarf^+/−^ mice at the ages as follows; Dwarf^+/+^ (10 male/8 female 11-14/9-34 months), Dwarf^+/−^ (5 male/4 female 11-14/12-14 months), respectively. **(G, H)** Circulating IGF-1 and insulin levels between Dwarf^+/+^ and Dwarf^+/−^ mice in males and females.

Supplementary Figure 2 Related to Heatmaps and Z-score averages expression data from RNA-seq of islets of βIgf1rKD and βIgf1r mice showing changes in Autophagy, G2-M DNA damage checkpoint, and DNA double-strand break response. Male and female at the age of 17-month-old, 4 mice per each group.

Supplementary Figure 3 Related to Metabolic changes under the five weeks of high-fat diet (HFD) of βIgf1rKD and βIgf1r or normal chow in βIgf1rKD mice; daily body weight and its AUC **(A, B)**, and food consumption of day 11 to 15 **(C, D)**.

Supplementary Table 1 The table of all the gene sequences used in qRT-PCR. Forward (F) and Reverse (R).
