# Supplementary figures and images for: Decreased IGF1R attenuates senescence and improves function in pancreatic β-cells
Source: Front Endocrinol (Lausanne). 2023 Jun 27;14:1203534. doi: 10.3389/fendo.2023.1203534 (PMC10335398; doi:10.3389/fendo.2023.1203534)

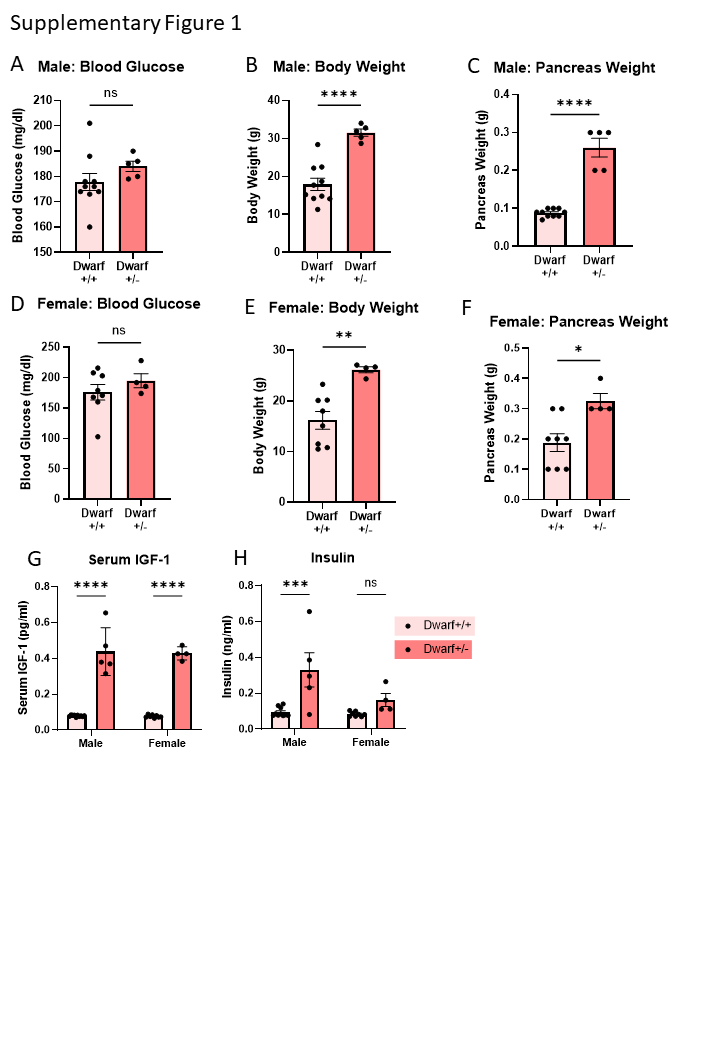

Supplement: Supplementary Figure 1 — Related to (A-F) The blood glucose level at 4 hours fast, body and pancreas weight were compared between Dwarf+/+ and Dwarf+/− mice at the ages as follows; Dwarf+/+ (10 male/8 female 11-14/9-34 months), Dwarf+/− (5 male/4 female 11-14/12-14 months), respectively. (G, H) Circulating IGF-1 and insulin levels between Dwarf+/+ and Dwarf+/− mice in males and females. [file Image_1.tif]

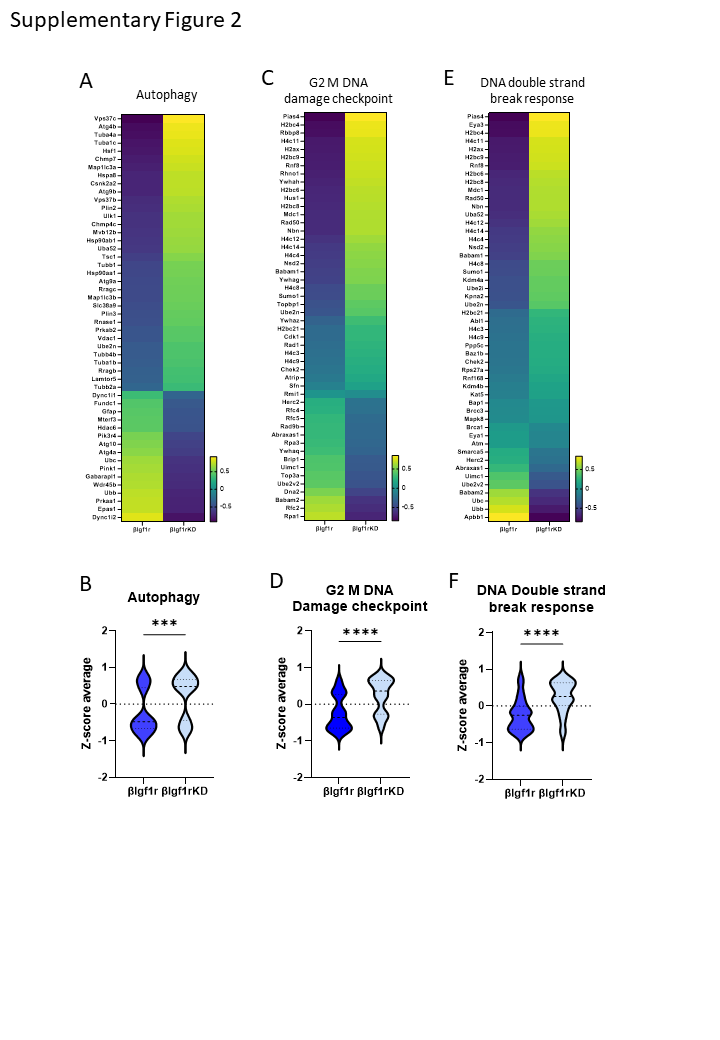

Supplement: Supplementary Figure 2 — Related to Heatmaps and Z-score averages expression data from RNA-seq of islets of βIgf1rKD and βIgf1r mice showing changes in Autophagy, G2-M DNA damage checkpoint, and DNA double-strand break response. Male and female at the age of 17-month-old, 4 mice per each group. [file Image_2.tif]

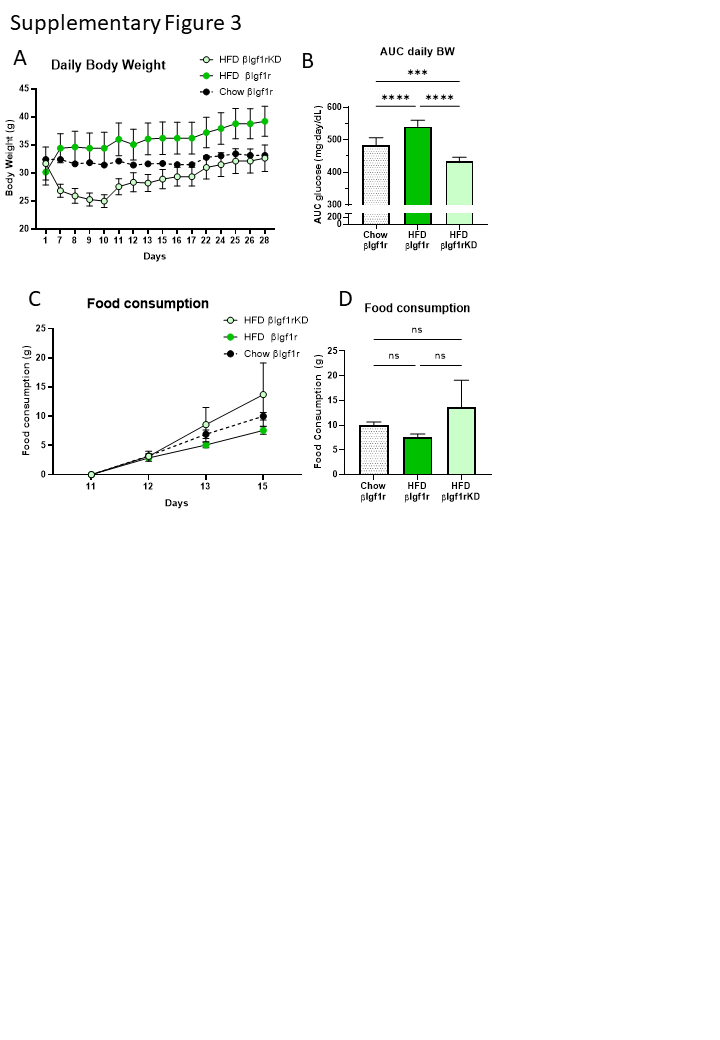

Supplement: Supplementary Figure 3 — Related to Metabolic changes under the five weeks of high-fat diet (HFD) of βIgf1rKD and βIgf1r or normal chow in βIgf1rKD mice; daily body weight and its AUC (A, B), and food consumption of day 11 to 15 (C, D). [file Image_3.tif]

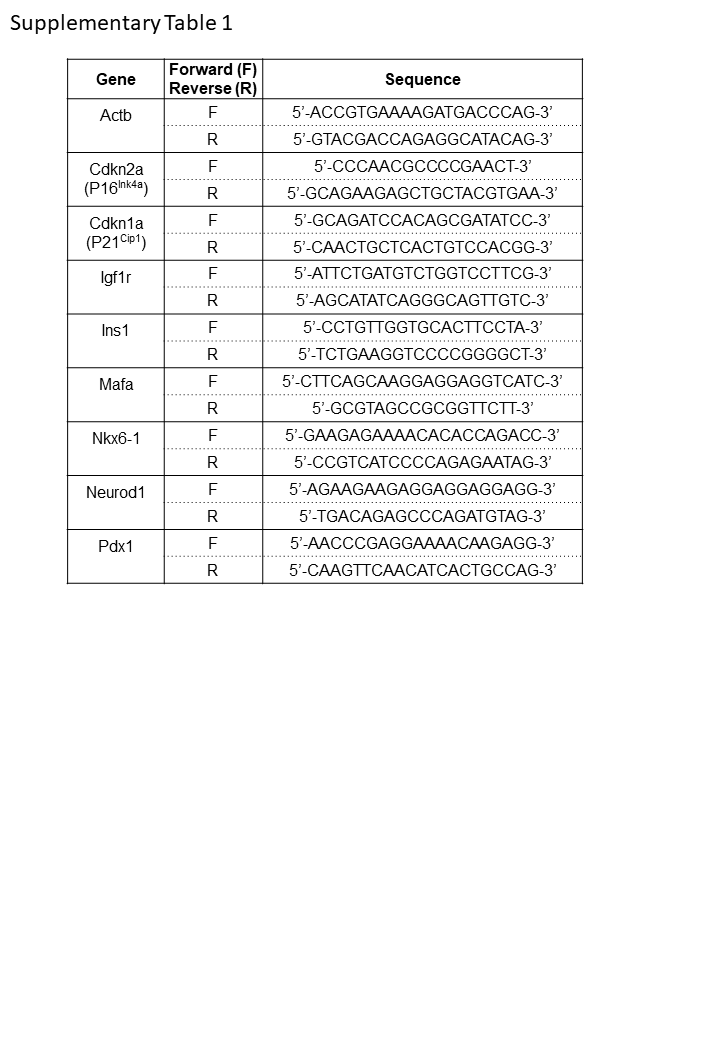

Supplement: Supplementary Table 1 — The table of all the gene sequences used in qRT-PCR. Forward (F) and Reverse (R). [file Table_1.docx]
